# Differential expression of virulence genes in *Legionella pneumophila* growing in *Acanthamoeba* and human monocytes

**DOI:** 10.1080/21505594.2017.1373925

**Published:** 2017-10-04

**Authors:** Qianqian Mou, Polly H. M. Leung

**Affiliations:** Department of Health Technology and Informatics, The Hong Kong Polytechnic University, Kowloon, Hong Kong, China

**Keywords:** *Acanthamoeba*, cell death, egress, *Legionella*, THP-1, virulence factors

## Abstract

*Legionella pneumophila*, the causative agent of Legionnaires’ disease, is widely distributed throughout natural and artificial water systems and can replicate in macrophages and amoebae. Amoebae are the natural hosts of *L. pneumophila*, whereas macrophages are incidentally infected. The life cycle of *L. pneumophila* comprises a replicative phase within the *Legionella*-containing vacuole (LCV) and a transmissive phase during which bacterial cells become motile and are released via killing of the host. Although the host death mechanisms induced by *L. pneumophila* have been studied, the expression patterns of related *L. pneumophila* genes have not been reported. The present study compared the expression patterns of host cell death-associated genes in *L. pneumophila* grown in the human monocytic cell line THP-1 and *Acanthamoeba castellanii*. Notably, when *L. pneumophila* was grown in THP-1, expression of the gene *flaA*, which is involved in the induction of pyroptosis, was downregulated during the course of infection. In contrast, *sdhA* associated indirectly with host death, was upregulated. Expression of the genes *vipD* and *sidF*, which are involved in the induction and suppression of apoptosis, changed by less than 2-fold. Notably, a lower percentage of pyroptotic cells was observed among infected THP-1 cells relative to uninfected cells, and the latter exhibited stronger expression of caspase-1. A different pattern was observed when *L. pneumophila* was grown in *A. castellanii*: *flaA* and *vipD* were activated, whereas *sdhA* and *sidF* were downregulated during the later stage of replication. The percentage of non-viable (annexin-V^+^ PI^+^ or annexin-V^+^PI^−^) *A. castellanii* organisms increased with *Legionella* infection, and the expression of metacaspase-1, which is involved in encystation was up-regulated at late infection time. In summary, *L. pneumophila* can multiply intracellularly in both amoebae and macrophages to induce cell death and secondary infection, and this characteristic is essential for its survival in water and the lungs. The gene expression profiles observed in this study indicated the increased cytotoxicity of *L. pneumophila* in *A. castellanii*, suggesting an increased adaptation of *Legionella* to this host.

## Introduction

*Legionella pneumophila* is the major causative agent of Legionnaires’ disease, a pneumonia-like illness associated with high mortality among immunocompromised populations.^1,2^ *L. pneumophila* is ubiquitously distributed throughout both natural and artificial water systems, including cooling towers, whirlpools, and shower heads which serve as important sources for pathogen transmission,^3,4^ and it can replicate in a wide range of hosts from protozoa to human macrophages.^5,6^ *Acanthamoeba* species are among the major environmental hosts of *Legionella*,^7^ and these organisms play key roles in the survival of *L. pneumophila* in aquatic environments.

The survival of *L. pneumophila* within different hosts hinges on successful evasion of the hosts’ conserved phagocytic killing pathways.^8^ The intracellular life cycle of *L. pneumophila* comprises replicative and transmissive (post-exponential) phases.^9^ During the replicative phase, *L. pneumophila* multiplies actively inside *Legionella*-containing vacuoles (LCV). Here, *L. pneumophila* effector proteins translocated through the Dot/Icm type IV secretion system (T4SS) can manipulate host cell functions and support intracellular growth in the LCV.^10,11^ After depleting nutrients inside the host, *L. pneumophila* enters the transmissive phase, wherein the organism becomes motile and achieves readiness for extracellular release via host cell death.^12^

In contrast to its standard intracellular replication strategy, *L. pneumophila* employs distinctive, host type-dependent mechanisms to induce host cell death. In mammalian macrophages, *L. pneumophila* induces cell death by apoptosis, a non-lytic, caspase-3-induced process, and pyroptosis, a lytic, proinflammatory process triggered by *L. pneumophila* flagellin and induced by host caspase-1.^13,14^ However, the mechanism of cell death induction in amoeba hosts is less well-defined. Although protozoan species possess a functional pathway that leads to apoptosis, it remains unclear whether this pathway is triggered by *L. pneumophila* infection.^15,16^ However, *Legionella*-infected amoeba hosts are known to undergo necrosis mediated by pore-forming activity.^17^ Furthermore, the release of *Legionella*-containing vesicles from the amoeba host was observed in *L. pneumophila*-infected hosts before encystation.^18^ Notably, this encystation process was mediated by metacaspase-1, a caspase-like enzyme.^19^ A more recent study reported that *L. pneumophila* infection reduced cell cycle protein production and impaired cell proliferation in the amoeba host.^20^

Key *L. pneumophila* virulence factors, including *flaA, vipD, sdhA*, and *sidF*, contribute to macrophage cell death.^21,22^ Of these, *flaA* encodes *L. pneumophila* flagellin, a flagellar component produced during the stationary growth phase of *L. pneumophila*.^23^ In addition to its role as a structural protein, flagellin exhibits effector functions: for example, the injection of flagellin into the host cytoplasm from the LCV leads to the activation of the inflammasome and caspase-1, and consequently to pyroptosis.^24^ Several researchers have studied the role of *vipD*. Gaspar *et al.* demonstrated that *vipD*, a phospholipase, could inhibit endosomal fusion with the LCV in COS-1 and CHO cell models, and observed rounding and death in VipD-producing cells.^25^ Additionally, Zhu *et al.* described *vipD*-induced apoptosis in *L. pneumophila*-infected macrophages, observing that this phospholipase hydrolyzed both phosphatidylethanolamine (PE) and phosphocholine (PC) to destabilize the mitochondrial membrane, and the consequent release of cytochrome c led to caspase-3 activation and apoptosis.^26^

In contrast to *flaA* and *vipD*, which trigger death in *L. pneumophila*-infected host cells, *sdhA* and *sidF* have been shown to inhibit host cell death. The gene *sdhA* encodes a Dot/Icm-translocated effector protein that prevents pyroptosis by blocking AIM2 inflammasome and caspase-1 activation and is therefore required for *L. pneumophila* multiplication within macrophages.^27^ In the absence of *sdhA* protein, *L. pneumophila* infection causes nuclear degradation, mitochondrial disruption, and significant cell death in infected macrophages.^28^ Additionally, a previous infection study reported an association of *sdhA*-deficient *L. pneumophila* with increased dendritic cell death.^29^ The *sidF* protein inhibits macrophage apoptosis by interacting with the endogenous pro-apoptotic Bcl-2 family proteins to facilitate the intracellular multiplication of *L. pneumophila*.^30^

These host cell death-related *L. pneumophila* genes directly affect the pathogen's intracellular replication cycle. Although studies have evaluated the roles of these genes in host cell death after *L. pneumophila* infection, no reported study has investigated trends in the expression of these genes in macrophages and amoebae during various stages of *L. pneumophila* multiplication. This study aimed to compare the expression of *flaA, vipD, sdhD, and sidF* during intracellular *L. pneumophila* replication and cell death in *Acanthamoeba castellanii* and THP-1 monocyte hosts.

## Results

### Determination of the post-exponential phase of L. pneumophila

**Figure 1.**
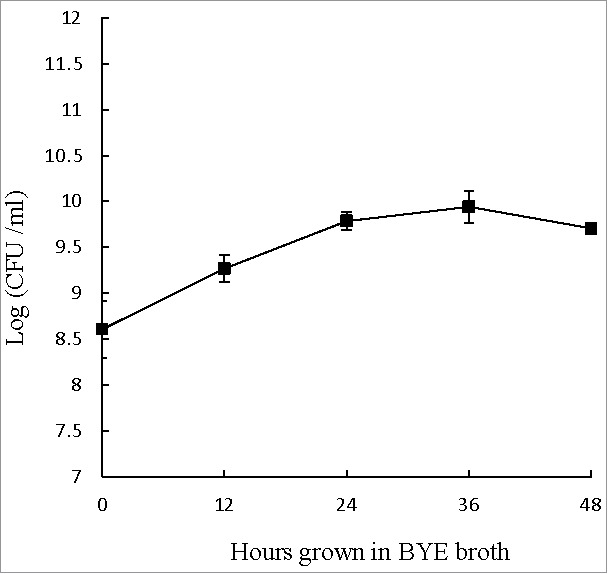
Extracellular growth curve of *L. pneumophila*. Number of viable and culturable *L. pneumophila* grown in BYE broth was enumerated at every 12 hours using plate count method. BCYE agar plates were used for plate count. Wilcoxon-signed rank test was used to compare the bacterial counts at 12 to 48 hours after inoculation with that at 0 hour. A *p*-value <0.05 was considered significant.

During extracellular growth in BYE broth, the number of *L. pneumophila* increased from 10^8^ to 10^10^ CFU ml^−1^ during the first 24 hours. From 24 to 48 hours, however, the numbers of viable *L. pneumophila* remained near 10^10^ CFU ml^−1^ (Fig. 1), suggesting that the bacteria might have reached the post-exponential phase (reportedly the most virulent phase) and would be ready to infect host cells.^31,32^ Accordingly, *L. pneumophila* cultured in BYE broth for 48 hours was used to infect THP-1 cells and *A. castellanii* in this study.

### Replication capacities of L. pneumophila in THP-1 cells and A. castellanii

**Figure 2.**
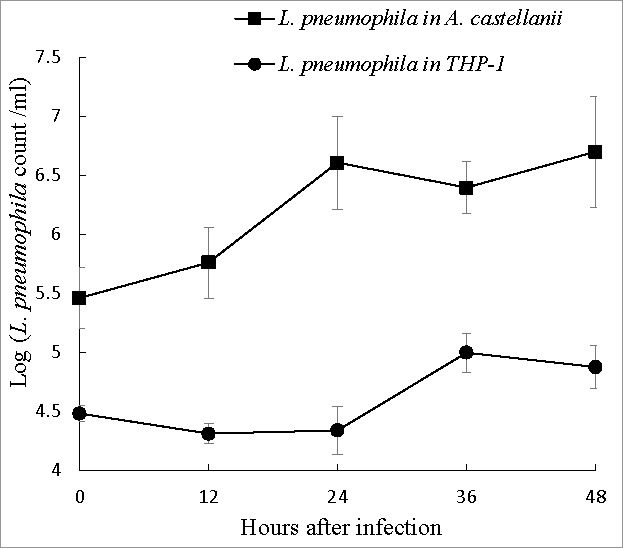
Intracellular growth of *L. pneumophila*. Viable and culturable *L. pneumophila* grown in THP-1 and *A. castellanii* were released by lysis of the host cells at 12-hour intervals up to 48 hours. The released *L. pneumophila* cells were enumerated using plate count method. BCYE agar plates were used for plate count. Wilcoxon-signed rank test was used to compare the bacterial counts at 12 to 48 hours after inoculation with that at 0 hour. A *p*-value <0.05 was considered significant.

THP-1 cells and *A. castellanii* were both challenged with *L. pneumophila* at a multiplicity of infection (MOI) of 10. Following gentamicin treatment, however, the initial intracellular *L. pneumophila* counts (reported as log CFU ml^−1^) at 0 hours were 4.48 in THP-1 cells and 5.54 in *A. castellanii*, which might indicate a difference in the hosts abilities to uptake *L. pneumophila*. When grown in THP-1 cells, the intracellular *Legionella* number remained stable during the first 24 hours post-infection, and exhibited a <1-log increase from 24 to 36 hours post-infection followed by another stable period from 36 to 48 hours (Fig. 2). By contrast, in *A. castellanii*, intracellular *L. pneumophila* exhibited a 1-log increase in growth during the first 24 hours post-infection, with no further increases up to 48 hours (Fig. 2), thus demonstrating better replication of *L. pneumophila* in *A. castellanii*.

### Expression of L. pneumophila virulence genes in different hosts

**Figure 3.**
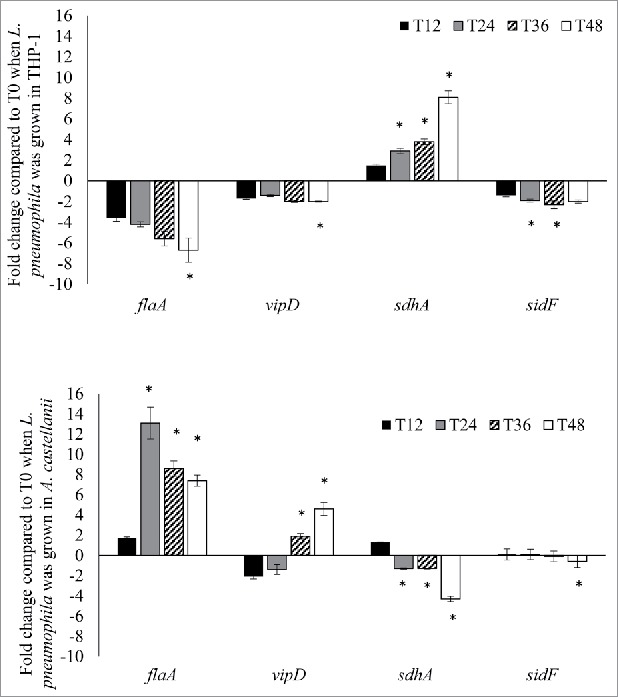
*L. pneumophila* virulence genes expression during intracellular growth in THP-1 and *A. castellanii. L. pneumophila* grown in THP-1 (upper panel) and *A. castellanii* (lower panel) at different time points were collected for RNA isolation and used to run quantitative RT-PCR. All virulence genes had been normalized to housekeeping gene *gyrB*. Data were expressed as the mean fold change (2^−ΔΔCT^) compared to T0. Error bars show the SEM (Standard error of the mean). Fold changes at T24 to T48 were statistically compared with that at T12 using Wilcoxon signed rank test, asterisks (*) represent significant differences (*p* < 0.05).

When grown in THP-1 cells, the expression of the pyroptosis-related genes *flaA* and *sdhA* varied more dynamically over time. The expression of *flaA* decreased from −3.6-fold to −6.7-fold over time, whereas the expression of *sdhA* was upregulated at all time points, increasing from a 1.5-fold upregulation at 12 hours to 8.1-fold at 48 hours post-infection (Fig. 3). By contrast, the expression of the apoptosis-related genes *vipD* and *sidF* was more static, with slight decreases (≤2-fold downregulation) over time (Fig. 3).

In *A. castellanii*, the expression of both *flaA* and *vipD* was upregulated. During the first infection cycle, *flaA* expression had not obviously changed (1.7-fold upregulation) at 12 hours post-infection, but peaked (13.1-fold) at 24 hours, followed by decreases during the second infection cycle to 8.6- and 7.4-fold upregulation at 36 hours and 48 hours, respectively. Although *vipD* exhibited 1.8- and 1.3-fold downregulation at 12 and 24 hours, respectively, the expression levels exhibited an increasing trend in the second infection cycle, with 1.8- and 4.6-fold upregulation at 36 and 48 hours, respectively (Fig. 3). By contrast, *sdhA* and *sidF* exhibited changes of <2-fold at most time points, except for a 4.3-fold downregulation of *sdhA* at 48 hours post-infection (Fig. 3). In summary, these gene expression studies indicate that during growth in THP-1 cells, *L. pneumophila* genes responsible for pyroptosis activation were downregulated, whereas those involved in suppressing host pyroptosis were activated. By contrast, the *L. pneumophila* genes responsible for initiating host cell death were upregulated during growth in *A. castellanii*.

### Expression of CASP genes in the host cells

**Figure 4.**
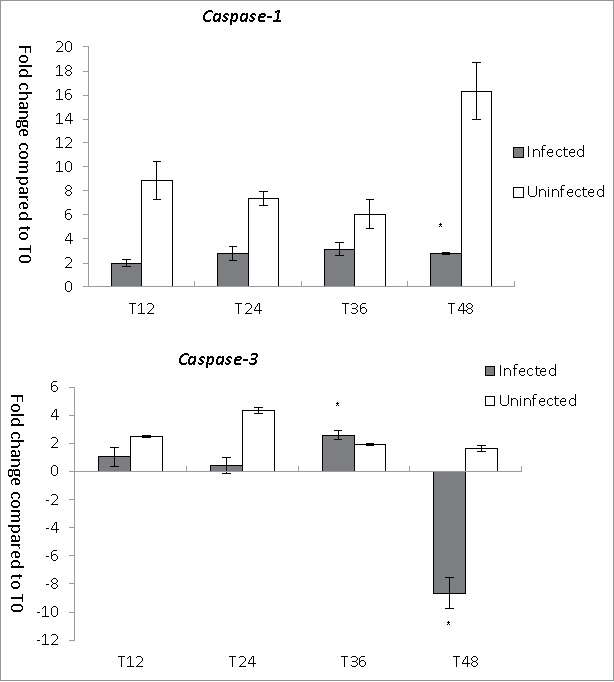
*CASP* genes expression during *L. pneumophila* infection in THP-1. Fold changes in the expression of *CASP*-*1* and *CASP-3* in THP-1 were detected using quantitative RT-PCR every 12 hours from T0 to T48. Expression levels of the THP-1 genes were normalized to those of its housekeeping gene *GAPDH*. Data were expressed as the mean fold change. Error bars show the SEM (Standard error of the mean). Fold changes at T24 to T48 were statistically compared with the that at T12 using Wilcoxon signed rank test, asterisks (*) represent significant differences (*p* < 0.05).

To understand the host cell responses during *L. pneumophila* infection, we investigated the expression of *CASP-1* and *CASP-3* in THP-1 cells (Fig. 4). In uninfected THP-1 cells, the expression of *CASP-1*, which encodes caspase-1, was strongly upregulated, with increases ranging from 6.1- to 16.3-fold over a period of 12–48 hours. Despite the strong upregulation of *CASP-1* at 48 hours, however, the viable cell number was not remarkably reduced. By contrast, in *L. pneumophila*-infected THP-1 cells, *CASP-1* was only modestly upregulated, with increases ranging from 2- to 3.2-fold between 12 and 48 hours post-infection. The expression of *CASP-3*, which encodes the apoptotic protein caspase-3, was lower in *L. pneumophila*-infected THP-1 cells than in uninfected cells at all time points except for T36, when the trend reversed slightly (Fig. 4). An 8.6-fold downregulation of *CASP-3* expression at 48 hours post-infection might have been caused by a decrease in the number of viable THP-1 cells. In summary, our results demonstrate reduced activation of caspase genes in the *Legionella*-infected cells relative to uninfected cells.

**Figure 5.**
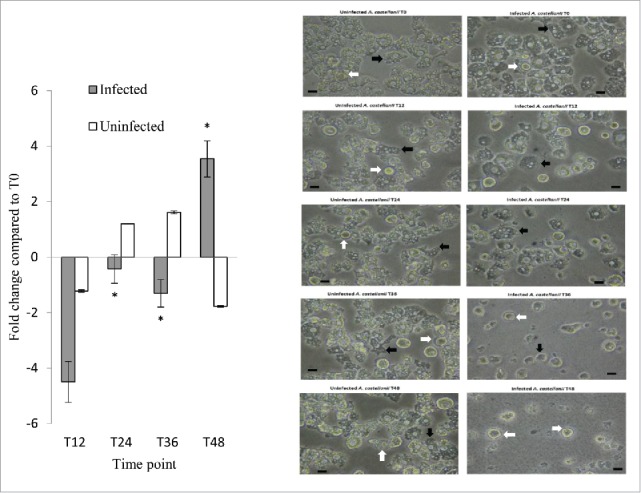
*MCASP-1* expression during *L. pneumophila* grown in *A. castellanii* that carried change from trophozoites to cysts. Fold changes in the expression of *MCASP-1* in *A. castellanii* were detected using quantitative RT-PCR every 12 hours from T0 to T48 (Left). The expression level of *MCASP-1* was normalized to that of *A. castellanii 18S rRNA* gene. Data were expressed as the mean fold change. Error bars show the SEM (Standard error of the mean). Fold changes at T24 to T48 were statistically compared with the that at T12 using Wilcoxon signed rank test, asterisks (*) represent significant change (*p* < 0.05). The microscopic images (Right) show the morphologies of the uninfected and infected *A. castellanii*. Black arrows indicate trophozoites and white arrows indicate cyst forms that have two-layer cell wall (scale bar = 25 μm, magnification 400 ×).

The expression of the *A. castellanii* metacaspase-1 gene (*MCASP-1*) was also investigated (Fig. 5, left panel). In uninfected *A. castellanii*, a <2-fold change in *MCASP-1* expression was observed from the 12- to 48-hour time points. In *Legionella*-infected *A. castellanii*, however, *MCASP-1* expression exhibited an increasing trend from 12 to 48 hours post-infection. At 12 hours, *MCASP-1* expression was downregulated by 4.5-fold, downregulated by only 1.5-fold at 24 and 36 hours, and upregulated by 3.5-fold at 48 hours, indicating that *MCASP-1* activation occurred at a later time point after *L. pneumophila* infection. As *MCASP-1* promotes amoeba encystment, we microscopically examined both uninfected and infected cells for the presence of amoeba cysts at different time points. At 48 hours, amoeba cysts were present in 1% and 32% of the uninfected and infected *A. castellanii*, respectively, demonstrating that *L. pneumophila* led to increased cyst formation (Fig. 5, right panel).

### Investigation of host cell death after L. pneumophila infection

We additionally evaluated the occurrence of cell death in the two hosts using annexin-V and propidium iodide (PI) staining. Positive double staining (annexin-V^+^PI^+^) served to indicate pyroptosis and late apoptosis, negative staining (annexin-V^−^PI^−^) indicated viability, annexin-V^+^ single positivity denoted early apoptosis, and PI^+^ single positivity indicated late necrosis or cell debris. In THP-1 cells, the pyroptosis/late apoptosis population (annexin-V^+^PI^+^) in the *Legionella*-infected and uninfected groups increased from 13.9% to 41.5% and from 8.33% to 53.9%, respectively, from 0 to 48 hours (Table 2), and significantly lower frequencies of this population were observed in the *Legionella*-infected group from 12 to 48 hours (*p* values <0.05). This corroborated the observed reduction in *CASP-1* expression in *Legionella*-infected cells. However, when we compared the percentages of viable cells and the trends of caspase gene expression, we noted that although the percentage of viable cells in the infected group was the lowest at 48 hours, the highest level of *CASP-1* expression was not observed at that time point. By contrast, a 16-fold increase in *CASP-1* expression was observed in the uninfected cells at 48 hours, even there was no remarkable corresponding decrease in the percentage of viablecells at T48 (Fig. 4). In summary, there was no clear relationship between the annexin-V/PI staining results and the caspase expression patterns.

The increased percentage of dead cells in the infected group at 0 hours might have resulted from experimental manipulation. Notably, both infected and uninfected THP-1 cells exhibited low frequencies of early apoptotic cells at all time points (<3% and 6%, respectively) (Table 2), although the infected group had significantly lower percentages at 24 and 48 hours (*p* values <0.05). The viable population decreased from 82.9% to 53.7% in the infected group and from 89.5% to 39.4% in the uninfected group over time (Table 2), and the infected group had a significantly higher percentage of viable cells relative to the uninfected group from 12 to 48 hours (*p* values <0.05). These results are further summarized in Tables 2 and 3.

As noted, protozoan species possess an apoptotic pathway, although it is not triggered by *L. pneumophila* infection.^16,17^ In our examination of *A. castellanii* viability, we observed that the annexin-V^+^PI^+^, or necrotic, population increased in both *Legionella*-infected and uninfected organisms from 0 to 36 hours (from 14.5% to 44.7% and from 1.61% to 39.4%, respectively) (Table 3). Notably, the percentage of necrotic cells was significantly higher in the *L. pneumophila-*infected population at all time points (*p* values <0.05). Furthermore, the percentage of annexin-V^+^PI^+^ cells decreased at 48 hours in both *L. pneumophila*-infected and uninfected *A. castellanii*, which might indicate the harvesting of the insufficient numbers of cells for flow cytometric analyses at the last time point. In contrast to THP-1 cells, the *L. pneumophila*-infected *A. castellanii* had higher percentages of necrotic cells, compared to the uninfected *A. castellanii* (*p* values <0.05). This increase in annexin-V^+^PI^+^ double-staining suggests the changes in host membrane permeability associated with necrosis.^17^ The percentages of early apoptotic cells (annexin-V^+^PI^⁻^) ranged from 4.1% to 6.5% and from 0.55% to 7.4% in *Legionella*-infected and uninfected *A. castellanii*, respectively (Table 3), and these percentages were significantly higher in *Legionella*-infected cells at 0 and 12 hours (*p* value <0.05). The viable populations (annexin-V^−^PI^^−^^) decreased from 59.1% to 33.8% in the infected group and from 72.4% to 39.1% in the uninfected group (Table 3), and the percentages of viable cells were significantly lower among infected *A. castellanii* relative to uninfected cells at all time points (*p* value <0.05). The strong reduction in viability among uninfected *A. castellanii* (72.4% at T0 vs. 39.1% at T48) might be attributable to a higher-than-optimal incubation temperature (37°C vs. the optimal 30°C).^33^

## Discussion

This study compared trends in the expression of *L. pneumophila* genes involved in the manipulation of host cell death. When *L. pneumophila* was grown in THP-1 cells, expression of the pyroptotic protein *flaA* was downregulated over time. By contrast, the expression of *sdhA*, which stabilizes the LCV and inhibits pyroptosis, increased steadily after *L. pneumophila* infection. The expression profiles of *flaA* and *sdhA*, together with the reduced expression of *CASP* genes in *L. pneumophila*-infected THP-1 cells, indicated that cell death pathways were inhibited after infection. Furthermore, the percentage of non-viable cells was higher in uninfected THP-1 cells than in *L. pneumophila*-infected cells. Although this inhibition of host death favored the intracellular multiplication of *L. pneumophila*, it might also hinder the egress of *L. pneumophila* and decrease the overall number of intracellular *L. pneumophila* in THP-1 cells. Our finding that *L. pneumophila* had a lower replication level when grown in THP-1 cells supports this possibility.

In a transcriptome study, Faucher *et al.* demonstrated the upregulation of *sidF* in *Legionella-*infected THP-1 cells.^34^ *Legionella sidF* is involved in phosphoinositide metabolism and remodeling, and is therefore essential to LCV maintenance.^35^ In our study, we observed a <2-fold downregulation of *sidF* expression at all time points. We similarly observed <2-fold changes in *vipD* expression levels. The involvement of both *sidF* and *vipD* in apoptosis suggests that pyroptosis, which involves pore formation and thus facilitates the release of intracellular agents, plays a more major role in THP-1 monocytes during *Legionella* infection. Despite the small changes in *vipD* and *sidF* expression, however, we observed an 8.6-fold downregulation of *CASP-3* expression at 48 hours post-infection that may have been caused by a decrease in the total number of viable THP-1 cells. Accordingly, this decrease might also have induced only a slight upregulation in *CASP-1* at 48 hours after infection.

In infected THP-1 cells, *flaA* downregulation occurred gradually, although significant changes in gene expression were observed at the late time point (48 hours) rather than earlier time points. The downregulation of *flaA* could be due to the low number of intracellular Legionella and slow consumption of nutrients that failed to induce *flaA* expression. *sdhA* was upregulated gradually over the time points studied, expression levels were significantly higher at 24 to 48 hours after infection. *sdhA* has an indirect role in inhibiting cell death by maintaining the integrity of LCV, an increased level of *sdhA* ensured protection of the *Legionella* replication niche.

In uninfected THP-1 cells, we observed a 16.3-fold upregulation of *CASP-1* expression at 48 hours, with no corresponding remarkable reduction in the viable cell number. Accordingly, we did not identify a clear relationship between the trends in viable cell numbers and *CASP-1* expression. Possibly, this was due to a decreased number of viable cells, particularly at 48 hours, as this would affect the RNA yields and quantified gene expression levels.

In this study, we observed a high percentage of non-viable cells among uninfected THP-1 cells. Previous studies have demonstrated crosstalk between pathways related to autophagy and other types of cell death.^36,37^ Growth in nutrient-deprived conditions causes starving mammalian cells to induce autophagy,^38,39^ which in turn induces mitochondrial dysfunction and activates apoptosis or inflammatory pyroptosis.^37,38^ The high rate of cell death among uninfected THP-1 cells might therefore be a result of nutrient deprivation.^39^ Although the infected THP-1 cells were also grown in a nutrient-poor environment, *L. pneumophila* might have suppressed host cell death during its replication phase inside the host.

Previous reports have not described the trends of *flaA, vipD, sdhA*, and *sidF* expression during a *L. pneumophila* infection of *A. castellanii*. In this study, we found that the expression patterns of these four genes differed somewhat from those in THP-1 monocytes. The growth of *L. pneumophila* in *A. castellanii* led to the upregulation of *flaA* and *vipD* and downregulation of *sdhA* at later time points. Similar to our results, Bruggemann *et al.* (2006) demonstrated an upregulation of *flaA* (*lpg1340*) from 4 to 11 hours post-infection during intracellular growth in *Acanthamoeba*.^40^ The activation of *flaA* is known to promote cell death. Our findings showed that *flaA* expression peaked at 24 hours post-infection during the intracellular growth phase of *L. pneumophila* in *A. castellanii*. This lined up with the pattern of pyroptosis observed in *Acanthamoeba*, which exhibited a peak at 24 hours post-*L. pneumophila* infection. This increase in host cell death after replication facilitates the egress of *Legionella* and further infection of new host cells.

Although we observed a sharp increase in *flaA* expression during the first infection cycle, this expression decreased and subsequently rebounded to a lower peak during the second cycle. This decrease in *flaA* corresponds to the invasion of new hosts and multiplication of *L. pneumophila* and may be attributable to the increased frequency of encystation. Furthermore, the second infection cycle was slower, leading to a subdued *flaA* response. Nevertheless, in infected *A. castellanii*, significant changes in *flaA* expression were observed at an early time point (24 hours), corresponding to the time point when *Legionella* had multiplied intracellularly to reach the point of release. Therefore, the *Legionella* genes responsible for controlling host cell death are better adapted to *Legionella* replication within *A. castellanii*, compared with THP-1 cells. Accordingly, *Legionella* species are better adapted to *Acanthamoeba* than to macrophages.

Although *vipD* expression was downregulated during the first infection cycle, the expression levels exhibited an overall increasing trend. Although the slow activation response is not fully understood, *vipD* might play a less important role relative to *flaA* in the regulation of *Legionella* growth within an *Acanthamoeba* host. In the second infection cycle, no obvious increase in the intracellular *L. pneumophila* count was observed, despite upregulated levels of *vipD*. During the second cycle, the proportion of *Acanthamoeba* cysts increases, and lysosomal proteases might degrade cytosolic proteins and organelles within these cysts.^41^ Notably, as *vipD* is an inhibitor of lysosome fusion, its upregulation might have reduced the fusion of lysosomes with LCVs during encystation. Regarding *sdhA*, the expression levels varied slightly from 12 to 36 hours, followed by a decrease at 48 hours. *sdhA* is involved in the stabilization of the LCV and protection from cell death. Accordingly, its downregulation would destabilize vacuoles, leading to cell death and the release of *L. pneumophila* from the host.

In our study, we observed an initial downregulation of *MCASP-1* expression in *Legionella*-infected *A. castellanii*, followed by a trend toward increased expression during the course of infection. In a previous study, bacteria-infected amoebae grown at lower temperatures exhibited upregulated expression of *MCASP-1* at 48 hours, compared to their uninfected counterparts.^18^ The protozoan metacaspase has been shown to activate encystment.^19,42,43^ During the course of infection, we observed cysts in both uninfected and *L. pneumophila*-infected *A. castellanii* at different time points, but note that more cysts were observed in the latter group at 24 hours and thereafter (Fig. 5).

Our findings regarding the intracellular growth of *Legionella* species in *Acanthamoeba* and THP-1 cells were similar to those reported by Weissenmayer *et al.* (2013)^44^ and Roland *et al.* (2013),^45^ respectively, but differed from those reported by Dey^46^ and Lebeau.^47^ We note that Dey reported the exponential replication of *Legionella* in *Acanthamoeba* only after 24 hours, while Lebeau observed *Legionella* duplication for up to 72 hours post-infection. The discrepancies in these findings could be due to differences in the experimental operations, including the MOIs, co-culture media,^31^ and methods used to lyse host cells.^48^

We note that our study of *Legionella* gene expression was subject to several limitations. First, the numbers of viable host cells decreased at later time points. Although we did not observe host cell exhaustion at late time points, a decrease in the number of viable host cells could affect the *Legionella* replication cycle, which might consequently affect gene expression. Second, growth of *A. castellanii* in the PYG media at later time points. Although amoebae can grow in PYG medium, our study used an incubation temperature of 37°C, which was not optimal for amoeba multiplication. However, we note that *Legionella* could not grow in PYG medium, which lacked a growth supplement. Third, the *Legionella* burdens (i.e., numbers of intracellular bacteria) differed between THP-1 cells and *A. castellanii*. To overcome this discrepancy, we normalized the expression of the four target genes to that of the reference gene *gyrB*, thus minimizing the effects of bacterial burden variations on the quantification of gene expression. Forth, accumulation of metabolic wastes in the culture environment could interfere with host viability, to minimize the background interference, uninfected cell controls have been set up in the study.

In summary, we demonstrated that the expression patterns of *L. pneumophila* genes involved in host cell death differ between THP-1 macrophage and *A. castellanii* hosts. Notably, in THP-1 cells, genes involved in cell death were downregulated while those involved in cell death suppression were upregulated, and the expression of the cell death-related genes *CASP-1 and -3* was lower in *L. pneumophila*-infected vs. uninfected THP-1 cells. However, the opposite expression pattern was observed in *A. castellanii* with regard to *L. pneumophila* genes, and a trend toward increased expression of the *A. castellanii MCASP-1* gene was observed in the *L. pneumophila*-infected group. Both *L. pneumophila* and *A. castellanii* are natural biofilm inhabitants, and our results demonstrate that the expression pattern of *flaA*, the *Legionella* gene responsible for inducing host death, was better adapted to the *Legionella* replication cycle within *A. castellanii*, compared to that within THP1 macrophages. Therefore, *Legionella* is better adapted to and more readily induces cell death in *A. castellanii*.

## Materials and methods

### Bacterial strains, acanthamoeba strain, and cell line used in this study

*L. pneumophila* Philadelphia strain (ATCC 33152) was used in this study. *L. pneumophila* was cultured on BCYE agar plates supplemented with αBCYE growth supplement and GVPC selective antibiotics (Oxoid, UK). The cultures were incubated at 37°C with 5% CO_2_ for 3–5 days. *L. pneumophila* was also cultured in liquid BYE broth according to previously described procedures.^49^

*A. castellanii* (ATCC 30234) was cultured in supplied PYG medium (2% peptone, 0.1% yeast extract, and 1.8% glucose^50^) at 25°C for 1–2 weeks.^51^ THP-1 cells (ATCC TIB-202) were were grown in RPMI 1640 growth medium (Gibco, USA) supplemented with 10% fetal bovine serum (Invitrogen, USA) and 1% penicillin/streptomycin (Gibco, USA) at 37°C with 5% CO_2_ for 3–5 days. Trypan Blue staining was used to enumerate viable cells. Briefly, 0.5 ml of a suspension of *A. castellanii* or THP-1 cells was mixed with 0.5 ml of 0.4% Trypan Blue stain, and the mixture was incubated at the ambient temperature for 5 min. Subsequently, the viable (i.e., unstained) cells were counted under a microscope using a hemocytometer.

### Extracellular growth of L. pneumophila for determination of the post-exponential phase

A liquid culture was used to determine the time required by *L. pneumophila* to reach the post-exponential phase. *L. pneumophila* colonies grown on BCYE plates were suspended in phosphate-buffered saline (PBS), and the absorbance was adjusted to 1.0 at an absorbance of 600 nm (A_600_). Five hundred microlitres of this bacterial suspension were inoculated into 10 ml of BYE broth, which was then incubated at 37°C for 48 hours with agitation at 250 rpm. One hundred microliters of BYE culture were withdrawn at 12-hour intervals for up to 48 hours to enumerate the bacterial density using the plate count method. All experiments were performed in triplicate. The post-exponential phase was defined as the time point at which there was no further increase in *L. pneumophila* growth.

### Intracellular growth assays

*L. pneumophila* was co-cultured with THP-1 cells at a MOI of 10.^31,52^ Briefly, 2 ml of a THP-1 cell suspension (10^6^ cells ml^−1^) were mixed with 20 μl of a post-exponential *L. pneumophila* suspension (10^9^ colony-forming units [CFU] ml^−1^). The mixture was centrifuged in a conical tube at 900 × g for 5 min to enhance bacterial contact with the THP-1 cells, which were then incubated at 37°C with 5% CO_2_ for 3 hours. After incubation, the pellet was washed once with PBS and incubated with 2 ml of gentamicin (100 μg ml^−1^) at 37°C for 2 hours to kill extracellular bacteria. Subsequently, the mixture was washed twice with PBS and re-suspended in 2 ml of fresh incomplete RPMI medium (without fetal bovine serum). This point was regarded as “time zero” (0 hours, T0). At T0 and at every 12-hour interval up to 48 hours (12 hours–48 hours, T12 – T48), 10^6^ THP-1 cells were incubated with 2 ml of sterilized distilled water at room temperature for 10 minutes. The THP-1 cells were then lysed by 5–10 forced passages through a 23-gauge syringe needle.^48^ *L. pneumophila* released from the lysed co-cultures were enumerated using the plate count method. The experiments were performed in triplicate.

A co-culture of *L. pneumophila* and *A. castellanii* was set up as described above for the co-culture of *L. pneumophila* and THP-1, except that PYG was used as the co-culture medium and Page's amoeba saline (PAS) was used to wash the co-culture contents.^50^ Two milliliters of *A. castellanii* suspension (10^6^ cells ml^−1^) were inoculated into each well of a 6-well plate, followed by the addition of 20 µl of *L. pneumophila* suspension (10^9^ CFU ml^−1^) to each well. After centrifugation at 900 × *g* for 5 min, the plate was incubated at 37°C with 5% CO_2_ for 3 hours. After a PAS wash, the medium in each well was replaced with 2 mL of gentamicin (100 μg ml^−1^). After 2-hour incubation, the co-culture mixtures were washed twice with PAS and re-suspended in 2 ml of PYG medium. This time point was regarded as T0. At every time point, 10^6^ *A. castellanii* were added to 2 ml of sterilized distilled water and incubated at room temperature for 10 minutes, followed by lysis via 5–10 forced passages through a 23-gauge syringe needle. The experiments were performed in triplicate.

### RNA isolation and reverse transcription

RNA samples were prepared from lysed co-cultures using the PureLink RNA Mini Kit (Invitrogen, USA) according to the manufacturer's instructions. A NanoDrop spectrophometer (Thermo Fisher Scientific, USA) was used to evaluate the purity and concentration of each isolated total RNA sample. A RNA sample was considered pure if the ratio of absorbance values at 260 nm and 280 nm (A260/280) fell within the range of 1.8–2.1. A consistent quantity of RNA (<800 ng RNA per DNase I reaction) was then digested using a DNaseI kit (Sigma, USA) at 37°C for 30 minutes to remove any DNA contamination. Subsequently, 10 µl of digested RNA were reverse transcribed into cDNA using a RevertAid First Strand cDNA Synthesis kit (Fermentas, USA). To monitor the presence of DNA contamination in the digested RNA sample, a second reverse transcription was without reverse transcriptase (no-reverse transcriptase control) was set up simultaneously.

### Quantitative real-time PCR to detect gene expression

The sequences of primers and probes are summarized in Table 1. Quantitative real-time PCR was performed in 20-µl PCR mixtures comprising 1 µl of cDNA, 1 × SYBR Green Mastermix (Roche, Germany), and 500 µM of each forward and reverse primer. For TaqMan assays, the 20-µl PCR mix comprised 1 µl of cDNA, 1 × TaqMan Universal PCR Mastermix (ABI, USA), and 1 µl of 20 × TaqMan probes (containing the probe and forward and reverse primers). PCRs were run on an ABI7500 system (Applied Biosystems, Foster City, CA). The thermal cycling conditions comprised an initial denaturation step at 95°C for 10 min, followed by 40 cycles of 94°C for 30 s and 60°C for 1 min. The threshold cycle (C_T_) values of the target genes were normalized to those of the reference genes. Fold changes in the expression of various target genes and T24 to T48 relative to those at T12 were compared using the Wilcoxon signed rank test. A *p* value <0.05 was considered significant.

**Table 1.** Primers and TaqMan probes used in this study. All primers and probes were designed using online Primer-BLAST program (National Centre for Biotechnology Information, US).

Gene	Primer/probe Sequence (5′ to 3′)	Amplicon size (bp)
Primers for *L. pneumophila*-specific genes		
*gyrB* (reference gene)	Forward primer: AGCGATGAATCAATTACCGT	123
	Reverse primer: ATCAAATTTACCTCCGGCAT	
*flaA*	Forward primer: GTTGCTGCTCCTCCTCCAAT	178
	Reverse primer: ATGGTTCTTTCTCTGGCGCA	
*sdhA*	Forward primer: ATCCAGAGCTTCTTGCGCTT	159
	Reverse primer: TACGCATCCAAACCCGTCAA	
*sidF*	Forward primer: GTTACAGGGCAGCCTGATGT	190
	Reverse primer: CCGCTTTTGCTTTGTCGGAA	
*vipD*	Forward primer: CAGCGCATGCACAAGCTATT	161
	Reverse primer: GAGGGCAAAGGCCTTCTCTT	
Primers and probe for *THP-1-*specific genes		
GAPDH gene (reference gene)	Forward primer: GACTCATGGTATGAGAGCTGG	205
	Reverse primer: TGGTCTGCAAAAGGAGTGAG	
*CASP-1*	Forward primer: CCTCCTCACAGTTGGGTAAT	225
	Reverse primer: GCAGCAGTGGTTCCTAAATG	
*CASP-3*	Forward primer: GATTATCCTGAGATGGGT	100
	Reverse primer: TTGCTGCATCGACATCTG	
	Probe: FAM-GGAATGACATCTCGGT- MGB	
Primers and probe for *A. castellanii* -specific genes		
18S rDNA gene (reference gene)	Forward primer: CTGCGAAAGCATCTGCCAAG	106
	Reverse primer: TGGTCGGCATCGTTTATGGT	
*MCASP-1*	Forward primer: CGTACACTCGATTTAGAAGC	100
	Reverse primer: CCCTGCTGGTATGGATCAGG	
	Probe: FAM-ATGGCATACCCCTACG-MGB

**Table 2.** Flow cytometric analysis of *L. pneumophila*-infected and uninfected THP-1 cells using annexin-V and PI-staining. THP-1 cells were harvested and stained with annexin-V and PI at 12-hour intervals up to 48 hours. Stained cell samples were analysed on a FACS Aria III flow cytometer and data were analysed using BD FACSDiva software. Paired t-test was used to compared the percentages of cells at various stages between *L. pneumophila*-infected and uninfected THP-1 cells. Asterisks (*) represent statistical significant differences (p < 0.05).

	% of cells at pyroptosis / late apoptosis (annexin-V^+^PI^+^)			% of cell at early apoptosis (annexin-V^+^PI^−^)			% of viable cells (annexin-V^−^PI^−^)		
Time point	Infected THP-1	Uninfected THP-1	*p* value	Infected THP-1	Uninfected THP-1	*p* value	Infected THP-1	Uninfected THP-1	*p* value
T0	13.9 ± 0.47	8.33 ± 0.65	0.0003*	1.93 ± 0.36	1.99 ± 0.6	0.947	82.9 ± 1.4	89.5 ± 1.31	0.001*
T12	19.6 ± 0.49	36.2 ± 0.85	0.00015*	2.68 ± 0.38	3.4 ± 0.75	0.062	76.2 ± 1.49	60.1 ± 1.8	0.00042*
T24	23.7 ± 0.88	40.1 ± 0.68	0.00005*	2.6 ± 0.77	3.48 ± 0.68	0.003*	72 ± 2.64	55.2 ± 2.03	0.00043*
T36	33.7 ± 1.10	53.7 ± 0.87	0.000044*	2.61 ± 1.10	3.75 ± 0.90	0.007*	62.3 ± 3.07	41 ± 1.82	0.002*
T48	41.5 ± 1.02	53.9 ± 0.66	0.00029*	2.53 ± 0.99	5.67 ± 0.63	0.004*	53.7 ± 3.07	39.4 ± 1.98	0.002*

**Table 3.** Flow cytometric analysis of *L. pneumophila*-infected and uninfected *A. castellanii* cells using annexin-V and PI-staining. *A. castellanii* cells were harvested and stained with annexin-V and PI at 12-hour intervals up to 48 hours. Stained amoeba samples were analysed on a FACS Aria III flow cytometer and data were analysed using BD FACSDiva software. Paired t-test was used to compared the percentages of cells at various stages between *L. pneumophila*-infected and uninfected *A. castellanii*. Asterisks (*) represent statistical significant differences (p < 0.05).

	% of cells at pyroptois / late apoptosis (annexin-V^+^PI^+^)			% of cell at early apoptosis (annexin-V^+^PI^−^)			% of viable cells (annexin-V^−^PI^−^)		
Time point	Infected *A. castellanii*	Uninfected *A. castellanii*	*p* value	Infected *A. castellanii*	Uninfected *A. castellanii*	*p* value	Infected *A. castellanii*	Uninfected *A. castellanii*	*p* value
T0	14.5 ± 0.08	1.61 ± 0.05	0.0000022*	6.35 ± 0.07	0.55 ± 0.03	0.00002319*	59.1 ± 0.24	72.4 ± 0.15	0.00002244*
T12	25.3 ± 0.06	10.8 ± 0.04	0.000001*	4.14 ± 0.05	1.42 ± 0.03	0.00003294*	36.6 ± 0.20	49 ± 0.14	0.00000819*
T24	40.9 ± 0.08	35.4 ± 0.06	0.00000304*	6.12 ± 0.06	7.41 ± 0.05	0.00002109*	26.6 ± 0.24	35.7 ± 0.19	0.00001657*
T36	44.7 ± 0.10	39.4 ± 0.07	0.00001408*	4.73 ± 0.10	5.69 ± 0.06	0.002*	26.1 ± 0.31	34.6 ± 0.22	0.00005129*
T48	36.8 ± 0.06	34.4 ± 0.07	0.00000738*	4.86 ± 0.04	5.2 ± 0.06	0.001_*_	33.8 ± 0.21	39.1 ± `0.24	0.0000729*

### Flow cytometric analysis of cell death using annexin-V and propidium iodide (PI) staining

A total of 10^6^ non-lysed, co-cultured cells (otherwise processed as described for intracellular growth assays) from both the *L. pneumophila*-inoculated and non-inoculated groups were collected and stained with fluorescein isothiocyanate (FITC)-labeled annexin-V and phycoerythrin (PE)-labeled PI (BD Pharmingen, USA). The labeled cells were then subjected to flow cytometry to determine the cell death frequencies. *A. castellanii* and THP-1 cells infected with *L. pneumophila* were collected at different time points (T0, T12, T24, T36 and T48), washed twice with ice-cold PBS, and incubated with 100 μl of 1 × staining buffer containing 5 μl annexin-V and 5 μl propidium iodide (PI). After a 15-minute incubation at room temperature in the dark, the reaction was stopped by adding 400 μl of 1 × annexin-V-stain buffer. The stained cells were analyzed on a FACS Aria™ III flow cytometer (BD, USA) on which the fluorescence compensation had been adjusted to minimize overlap of the FITC and PE signals. A total of 50,000 events were acquired for each samples. The flow cytometry data were analyzed using BD FACSDiva Software (BD, USA). The experiments were performed in triplicate, and data were presented as percentage. *L. pneumophila*-inoculated and non-inoculated groups were compared using paired t-test, and p value <0.05 was considered significant.
